# Oiling the Brain: A Review of Randomized Controlled Trials of Omega-3 Fatty Acids in Psychopathology across the Lifespan

**DOI:** 10.3390/nu2020128

**Published:** 2010-02-09

**Authors:** Natalie Sinn, Catherine Milte, Peter R. C. Howe

**Affiliations:** Nutritional Physiology Research Centre, Sansom Institute for Health Research, University of South Australia, Frome Road, Adelaide 5000, Australia; Email: catherine.milte@unisa.edu.au (C.M.); peter.howe@unisa.edu.au (P.H.)

**Keywords:** omega-3 polyunsaturated fatty acids, mental health, attention deficit hyperactivity disorder, autism, aggression, depression, dementia

## Abstract

Around one in four people suffer from mental illness at some stage in their lifetime. There is increasing awareness of the importance of nutrition, particularly omega-3 polyunsaturated fatty acids (n-3 PUFA), for optimal brain development and function. Hence in recent decades, researchers have explored effects of n-3 PUFA on mental health problems over the lifespan, from developmental disorders in childhood, to depression, aggression, and schizophrenia in adulthood, and cognitive decline, dementia and Alzheimer’s disease in late adulthood. This review provides an updated overview of the published and the registered clinical trials that investigate effects of n-3 PUFA supplementation on mental health and behavior, highlighting methodological differences and issues.

## 1. Introduction

Mental health is described as “a state of well-being in which every individual realizes his or her own potential, can cope with the normal stresses of life, can work productively and fruitfully, and is able to make a contribution to her or his community” [1]. About 450 million people worldwide do not enjoy this state of wellbeing, and it is estimated that one in four people will suffer from mental illness at some stage in their lifetime, causing enormous suffering, personal, social and financial burden, as well as associated physical illness [2]. Identification of preventable risk factors and effective treatments therefore constitutes an international priority.

Nutrition has an important role in mental health as the brain relies on both macro- and micronutrients for development and functioning [3,4,5,6]. In particular, suboptimal levels of omega-3 polyunsaturated fatty acids (n-3 PUFA) are emerging as a potentially modifiable risk factor for mental illness. Since their discovery in the 1970s as key components of brain tissue [7], long-chain n-3 PUFA have been postulated to serve critical roles in brain development, and function and a lack of these fatty acids has been implicated in a number of mental health conditions over the lifespan, from developmental disorders and mental retardation in childhood, to depression, bipolar disorder, schizophrenia and borderline personality disorder, stress, hostility and aggression in adulthood, and cognitive decline, dementia and Alzheimer’s disease in late adulthood [8]. 

Although the etiology of these disorders is clearly multifactorial and subject to individual variation, the number of clinical trials addressing suboptimal n-3 PUFA levels as a possible underlying biological contributor to associated symptoms, and importantly symptoms that often co-occur and overlap between these disorders, is steadily growing. Following an overview of n-3 PUFA, we will provide a review of randomized controlled trials [8], and an update of recent trials located via the World Health Organization’s International Clinical Trials Registry Platform Search Portal [9], including preliminary results from our own recent studies, focusing on the role of n-3 PUFA in alleviating symptoms of psychopathology from childhood throughout adulthood. Trial identification numbers of registered trials not included in the original review were searched on PubMed to check if they had been published. Published and registered trials are summarized in Table 1 and Table 2, respectively.

## 2. Omega-3 PUFA

### 2.1. Overview

The omega-6 (n-6) and n-3 families of PUFA have numerous biological roles in cellular structure and function. Alpha linolenic acid (ALA; 18:3n-3), parent n-3 PUFA, and linoleic acid (LA; 18:2n-6), parent n-6 PUFA, cannot be synthesized by humans and must be provided via the diet. They are therefore termed essential fatty acids (EFA). Humans can obtain ALA in their diet via plant sources. EFA have 18 carbon atoms with double carbon bonds. These chains undergo further processes of elongation and de-saturation (insertion of additional double bonds) to form highly unsaturated long chain (LC) PUFA. Our bodies can perform these conversions, although not always very efficiently. Long chain (LC) derivatives of ALA, eicosapentaenoic acid (EPA) and docosahexaenoic acid (DHA), are made by phytoplankton and transferred via the food-chain to marine organisms, particularly cold-water fish and marine animals. Therefore, humans can also obtain these elongated n-3 PUFA directly via algal or fish sources. 

Structurally, PUFA are key components of phospholipids, comprising cellular and intracellular membranes. They govern growth and vitality through oxidation (metabolism of food to produce energy required for cellular processes), chemical activities and transportation. As well as constructing membranes and generating and propagating electrical impulses, LC-PUFA are required for synthesis of eicosanoids, important signaling hormones with numerous complex functions. Those derived from n-3 LC-PUFA are generally anti-inflammatory, anti-thrombotic and vasodilatory, balancing and counteracting pro-inflammatory, vasoconstrictor actions of eicosanoids derived from n-6 LC PUFA [10]. The cardiovascular benefits of n-3 PUFA [11] are largely attributed to these eicosanoid properties, and they may also have important roles in brain function.

### 2.2. Omega-3 PUFA and the Brain

Lipids constitute approximately sixty percent of the dry weight of the brain. DHA and AA are the most highly concentrated PUFA in neural phospholipids, including subcellular membranes [12,13,14]. Although the circulation contains at least 10 times more n-6 than n-3 PUFA, DHA predominates in the retina, brain and nervous system [14]. Furthermore, DHA is particularly concentrated at neural synapses [15,16,17,18,19].

Importantly, DHA levels in neural membranes vary according to dietary oil intake [4,19,20] and there is evidence that low PUFA and high cholesterol levels reduce phospholipid fluidity [19,20]. Animal studies and human infant autopsies have also shown that when insufficient n-3 PUFA are available during early neural development, there is a decrease in the DHA content of the brain. Furthermore, increased n-6 to n-3 PUFA ratios can also alter the fatty acid composition of neural membranes [21]. 

Various reviews have outlined biological mechanisms for n-3 PUFA in brain function [22,23,24]. In particular, and in line with a longstanding focus on the role of neurotransmitters such as dopamine and serotonin in mental illness, research has focused on associations between PUFA and central nervous system activity [25]. Evidence points to the role of eicosanoids in healthy brain functioning, and phospholipid membranes in neural cell signaling [12,19,26,27]. Levels of n-3 PUFA have been associated with monoaminergic neurotransmitter levels [27,28,29,30]. n-3 deficiency has also been found, in animal studies, to reduce levels of phosphatidylserine (PS) in the brain, which is thought to play an important function in neural signaling activities [12]. In alcoholics, DHA deficiency has predicted reduced 5-hydroxyindoleacetic acid (5-HIAA) concentrations in cerebrospinal fluid, an indicator of low serotonin turnover rate in the frontal cortex [31]. 

Studies have further indicated that n-3 PUFA may affect receptor properties or activation of signal transduction by receptors [30]. Electrical impulse conduction is dependent on the exchange of ions through the cell membrane, which relies on the fluidity and physiological structure of cell membranes. Furthermore, there are indications that PUFA are involved in the synthesis and activities of brain peptides [19], which are involved in modulating the activities of neurotransmitters [32]. n-3 PUFA are also thought to influence gene expression of a range of enzymes required for important neural functions including synaptic transduction, ion channel formation, energy metabolism and formulation of proteins vital for brain development and function [22]. 

Regular delivery of oxygen and nutrients via the blood is also critical for optimal brain function, and psychopathology is associated with both reduced cerebral blood flow and transportation of glucose, the brain’s primary energy source, to brain regions as required. In this regard, n-3 PUFA are associated with production of nitric oxide [33], as well as anti-inflammatory and vasodilatory eicosanoids (notably PGI_2_), and are known to assist in endothelial-dependent vasodilation [10]. They have also been associated with substantially increased transport of glucose across the blood-brain barrier [34,35]. Therefore, it is also possible that their primary influence on brain function includes improved cerebral blood flow and blood-brain barrier integrity [36]. This notion is supported by the high co-morbidity between cardiovascular disease and psychopathology, indicative of a common underlying vascular pathology that may be mediated by lifestyle factors such as suboptimal levels of n-3 PUFA [37].

A review of controlled animal studies [30] concluded that there is sufficient evidence of a role for n-3 PUFA in improving learning and behavior. A large body of research has investigated n-3 PUFA in infant development, which is also reviewed elsewhere [38]. The present review will focus on mental health problems in childhood through to late adulthood.

## 3. PUFA and Mental Illness

PUFA deficiencies have been reported in people with a range of psychiatric problems [22,23,24,39,40,41,24,39], including developmental disorders such as attention deficit hyperactivity disorder (ADHD), depression, bipolar disorder, stress, aggression, hostility and criminality, and dementia. A small but growing body of clinical research trials has been conducted with variable outcomes. These will be reviewed here, and are summarized in Table 1.

**Table 1:** Double-blind randomized placebo-controlled trials of omega-3 fatty acids in mental illness.

Authors	Participants	Daily dosage	Length of trial	Measures	Outcomes*
Voigt *et al.* (2001) [53]	*N* = 54; 6-12 year old (78% boys); idiopathic ADHD diagnosis; were being treated successfully with medication	345 mg DHA (algae-derived) or undefined placebo	16 weeks	CPRS; CBC; TOVA; CCT	Treatment = placebo on all measures
Stevens *et al*. (2003) [59]	*N* = 50; 6-13 year old (78% boys); ADHD diagnosis; high FADS^l^; some on medication (equally allocated to conditions)	96 mg GLA, 40 mg AA, 80 mg EPA, 480 mg DHA, 24 mg Vit E or olive oil placebo	16 weeks	DBD; ASQ; CPT; WJPEB-R; FADS	*Treatment > placebo: DBD-Conduct (parents); DBD-Attention (teachers). *Other 14 outcome measures non-significant
Hirayama *et al.* (2004) [55]	*N* = 40; 6-12 year old (80% boys); ADHD diagnosis; 15% medicated; 82% comorbid conditions	100 mg EPA, 514 mg DHA or olive oil placebo (supplied in soymilk & bread)	8 weeks	ADHD DSM-IV; DTVP; STM; CPT; Other	Treatment = placebo on all measures (except that placebo > treatment on CPT and STM)
Richardson & Puri (2002) [58]	*N* = 29; 8-12 year old (62% boys); normal IQ; low reading ability; above average ADHD scores on Conners’ Index; no participants in treatment for ADHD	864 mg LA, 42 mg AA, 96 mg ALA, 186 mg EPA, 480 mg DHA, 60 iµ Vit E or olive oil placebo	12 weeks	CPRS	*Treatment > placebo: CPRS; Cognitive problems/inattention; Anxious/shy; Conners’ global index; DSM inattention; DSM hyperactive/impulsive; Conners’ ADHD Index*
Richardson & Montgomery (2005) [60]	*N* = 117; 5-12 year old (77% boys); Developmental Coordination Disorder, 1/3 with ADHD symptoms in clinical range, not in treatment; IQ > 70;	60 mg AA, 10 mg GLA, 558 mg EPA, 174 mg DHA, 9.6 mg Vit E or olive oil placebo	12 weeks active *vs.* placebo; one-way crossover to active treatment for 12 weeks	MABC; WORD; CTRS	*Treatment > placebo: WORD; CTRS Oppositional behaviour; Cognitive problems/inattention; Hyperactivity; Anxious/shy; Perfectionism; Social problems; Conners’ index; DSM-IV inattention, hyperactive/impulsive *
					Treatment = placebo: MABC
Sinn *et al.* (2007; 2008) [61,62]	*N* = 132 (questionnaire data available for 104); 7-12 year old (74% boys); ADHD symptoms in clinical range; unmedicated	60 mg AA, 10 mg GLA, 558 mg EPA, 174 mg DHA, 9.6 mg Vit E, or palm oil placebo	15 weeks active *vs.* placebo; one-way crossover to active treatment for 15 weeks	CPRS, CTRS Vocabulary, subtests from WISC-III & TEA-ch, Stroop	*Treatment > placebo CPRS: Cognitive problems/inattention; Hyperactivity; ADHD Index; Restless/Impulsive; DSM-IV Hyperactive/Impulsive; Oppositional. *Treatment = placebo on other subscales and CTRS.
					*Treatment > placebo on Creature Counting & vocabulary. *Treatment = placebo on other cognitive tests
Johnson *et al.* (2008) [64]	*N* = 75, 8-18 year old children with diagnosed ADHD, unmedicated (85% males)	60 mg AA, 10 mg GLA, 558 mg EPA, 174 mg DHA, 9.6 mg Vit E or olive oil placebo	3 months active *vs.* placebo; one-way crossover to active treatment for 3 months	Investigator-rated ADHD Rating Scale-IV; CGI	Treatment = placebo overall *Treatment > placebo in subgroups with inattentive subtype & comorbid neurodevelopmental disorders*
Milte *et al.* (in preparation) [65]	*N* = 54 (45 with bloods), 7-12 year old (79% male) with ADHD/ADHD symptoms (50% diagnosed) ACTRN012607000332426	1g EPA-rich oil, 1g DHA rich oil or sunflower oil placebo	3 x 3 crossover (4 months on each treatment)	CPRS, reading, writing, vocabulary, TEA-ch	Treatment = placebo in 12-month crossover.
					*Over 4 months erythrocyte DHA increases associated with improvements on CPRS - oppositional behaviour, anxiety/shyness – divided attention & reading. In subgroup with learning difficulties (n = 16 with blood) also on CPRS hyperactivity/impulsivity and spelling.*
Amminger *et al*. (2007) [80]	*N* = 13 (5-17 years) with Autistic Disorder (81.9% male)	1.5g/d n-3 PUFA (0.84g EPA, 0.7g DHA), Vit E ; or coconut oil placebo	6 weeks parallel design	Aberrant Behavior Checklist (ABC)	*Treatment > placebo for stereotypy & hyperactivity (trends with large effect sizes) *
					Treatment = placebo on 3 other subscales
Peet & Horrobin (2002) [103]	*N *= 70 (18-70 years), depressed (>15 on HDRS), medicated	Ethyl-EPA – 1, 2 or 4 g/day or placebo	12 week parallel design, adjunctive therapy	HDRS, MADRS, BDI	*Treatment > placebo on all 3 rating scales with 1g/day EPA – strong effects for core depressive symptoms. *Treatment = placebo on 2g and 4g/day (non-significant trends)
Nemets *et al*. (2002) [104]	*N* = 20 (28-73 years), diagnosed major depression disorder (85% women) HDRS score > 18	2g ethyl-EPA (96% from fish oil) or placebo, Vit E	4 weeks parallel design, adjunctive therapy	HDRS	*Treatment > placebo at weeks 2, 3 and 4 on HDRS score and on core depressive symptom subscales.*
Su *et al.* (2003) [106]	*N *= 22 (18-60 years), outpatients with major depressive disorder; HDRS score > 18, medicated	3.3g/day n-3 PUFA (2.2g DHA, 1.1g EPA)	8 weeks parallel design, adjunctive therapy	HDRS	*Treatment > placebo on HDRS*
Marangell *et al*. (2003) [111]	*N *= 35 (18-65 years), major depressive disorder diagnosis; HDRS score > 16 (80% female)	2g/day DHA or placebo	6 weeks parallel	MADRS, HDRS, GAFS	Treatment = placebo on outcome measures
Silvers *et al.* (2005) [112]	*N *=77 (18-65 yrs recruited, mean age 38), being treated for current depressive episode (53% female)	3g/day n-3 PUFA (2.4g DHA ; 0.6g EPA) + Vit E or olive oil placebo	12 weeks parallel, adjunctive therapy	HDRS short form, BDI	Treatment = placebo on outcome measures (improvements in both groups at week 2)
Nemets *et al.* (2006) [105]	*N *= 20 (6-12 year old; 25% girls); children with major depressive disorder	400mg EPA + 200mg DHA/day or safflower oil/olive oil placebos	16 weeks, parallel (5 children medicated)	^a^CDRS, CDI, CGI	*Treatment > placebo on outcome measures*
Grenyer *et al.* (2007) [113, 114]	*N = *83(18-72 years, *M* = 45)*, *outpatients with major depression diagnosis	3g/day n-3 PUFA (2.2g DHA, 0.6g EPA) + Vit E or olive oil placebo	4 month parallel design, adjunctive therapy	HDRS, BDI, GAFS	Treatment = placebo on outcome measures (improvements in both groups)
Su *et al.* (2008) [109]	*N *= 24 (18-40 years); with major depressive disorder during pregnancy	2.2g EPA + 1.2g DHA or placebo, both with tocopherols & orange flavor	8 weeks, parallel design	HDRS, EPDS, GDI	*Treatment > placebo on outcome measures*
Rogers *et al*. (2008) [117]	*N = *190 (18-70 years recruited, mean age = 38); people from GP surgeries or public with mild-moderate depression (77% female)	630mg EPA, 850mg DHA, 870mg olive oil, or olive oil placebo (both with tocopherols & orange oil)	12 weeks parallel design	DASS, BDI, STAEI, mood using diary and visual probe task, cognitive function	Treatment = placebo on outcome measures (improvements in both groups)
Van de Rest *et al.* (2008) [118]	*N* = 302 (> 65 years, *M* = 70; 55% male) non-depressed community dwelling adults. NCT00124852	1.8g/day EPA + DHA, 400mg/day EPA + DHA or placebo	26 weeks parallel design	CES-D, MADRS, GDS-15, HADQ, [POMS short form (*n* = 104)]	Treatment = placebo on outcome measures
Freund-Levi *et al.* (2008) [115]	*N* = 204 (mean age 73 years); people with AD living in own homes, on stable treatment with acetylcholine esterase inhibitors. NCT00211159	1.72g DHA + 600mg EPA/day or corn oil placebo	6 months parallel + one-way crossover to fish oil for 6 months	NPI, MADRS, CGB, DAD	Treatment = placebo on outcome measures. *Treatment > placebo on MADRS in non-apoE-4 carriers and agitation in apoE-4 carriers*
Lucas *et al.* (2009) [108]	*N = *120 (recruited 40-55 yrs; mean age 49) post-menopausal women with psychological distress & depressive symptoms	1.5g ethyl-EPA, 0.5g ethyl-DHA	8 weeks parallel design	PGWB, HSCL-D-20, HDRS	Treatment = placebo on all measures (improvements in both groups). *Treatment > placebo in women without MDE (major depressive episode diagnosis)*
Carney *et al.* (2009) [116]	*N = *122; major depression + coronary heart disease NCT00116857	930mg ethyl-EPA + 750mg ethyl DHA/day or corn oil placebo	10 weeks parallel design, adjunctive therapy	BDI-II; HDRS	Treatment = placebo on outcome measures (improvements in both groups)
Stoll *et al.* (1999) [120]	*N = *30 (18-65 years); inpatients with bipolar disorder	9.6g/day n-3 PUFA (6.2g EPA, 3.4g DHA) or olive oil esther placebo	4 month parallel design; adjunctive therapy	HDRS, YMRS, CGI-S, GAS	*Treatment > placebo on GAS, HDRS and CGI;* Treatment = placebo on YMRS
Keck *et al*. (2006) [123]	*N = *116 (n= 57 bipolar depressed; n = 59 rapid cycling), mean age: 45; 51% male	6g/day ethyl-EPA or liquid paraffin placebo	4 month parallel design; adjunctive therapy	IDS, YMRS, CGI-BP (bipolar disorder)	Treatment = placebo on outcome measures
Frangou *et al.* (2006) [124]	*N* = 75 (mean age: 47); outpatients with bipolar depression + scores > 17 on HDRS (76% female)	1g/day ethyl EPA (n = 24) ; 2g/day ethyl EPA (n = 25) or paraffin placebo	12 week parallel design, adjunctive therapy	HDRS, YMRS, CGI	*Treatment > placebo on HDRS & CGI on 1g and 2g/day. *Treatment = placebo on YMRS
Hallahan *et al.* (2007) [127]	*N *= 49 (16-64 years, *M* = 30); presenting after act of repeated self-harm (65% women)	1.2g/day EPA + 0.9g DHA or corn oil placebo (with 1% EPA/DHA)	12 weeks parallel design in addition to standard care	BDI, HDRS, OAS-M, IMT/DMT, PSS, DHUS	*Treatment > placebo on BDI, HDRS, PSS, DHUS. *Treatment = placebo on OAS-M & IMT/DMT (hostility/aggression, memory)
Peet *et al.* (2001) [139]	Study 1: *N* = 45 (mean age: 44 yrs); schizophrenic patients, PANSS score > 40	2g EPA, 2g DHA or placebo	3 months parallel adjunctive therapy	PANSS	*EPA treatment > placebo or DHA on positive PANSS score. *Treatment = placebo on negative symptoms score
	Study 2: *N* = 30 (mean age 35 years); diagnosed schizophrenia, untreated	2g/day EPA or corn oil placebo	3 months parallel, single therapy unless drugs needed	PANSS; need for antipsychotic medication	*Treatment > placebo, particularly on positive subscale; 12/12 placebo and 8/14 EPA patients took medication *
Fenton *et al*. (2001) [141]	*N *= 87 (18-65 years, *M* = 40; 61% male) diagnosed schizophrenia or schizoaffective disorder	3g/day ethyl EPA + Vit E or mineral oil + Vit E placebo	16 week parallel design, adjunctive therapy	PANSS, CGI, MADRS, RBANS, AIMS, SARS	Treatment = placebo on outcome measures (some showed improvements in both groups)
Emsley *et al.* (2002) [138]	*N *= 40 (18-55 years, *M* = 45); schizophrenic, treatment resistant patients, PANSS score > 10	3g/day ethyl-EPA or liquid paraffin placebo	12 weeks parallel design, adjunctive therapy	PANSS, ESRS	*Treatment > placebo on PANSS and dykinesia subscale of ESRS. *Treatment = placebo on other ESRS subscales
Peet & Horrobin (2002) [140]	*N *= 115 (20-62 years, *M* = 37; 66% male), treatment-resistant schizophrenia; PANSS > 50	1, 2 or 4g/day ethyl-EPA or liquid paraffin placebo	12 weeks parallel design, adjunctive therapy	PANSS, LUNSERS, MADRS, AIMS, BAS, SARS	Treatment = placebo on all rating scales; *2g treatment > placebo for patients on clozapine (associated with ↑AA)*
Berger *et al*. (2007) [144]	*N *= 69 (mean age 21 ± 4; 76% male) first episode psychosis patients	2g/day ethyl-EPA or mineral oil placebo not absorbed by intestinal tract (both with Vitamin E)	12 weeks parallel design, adjunctive therapy	BPRS, SANS, CDSS, CGI, GAF, SOFAS	Treatment = placebo on all outcome measures.
					*Treatment > placebo on CGI co-varying for duration of untreated psychosis; treatment > placebo at weeks 4-6*
Amminger *et al.* (2010) [145]	*N *= 81 (13-25 years, M = 16 ± 2, 40% male), met defined risk factors for psychosis				
Hamazaki *et al*. (1996) [147]	*N = *41 (19-30 years, 70% female); university students (study measured aggression and executive function)	1.5-1.8g/day DHA or 97% soybean oil + 3% fish oil placebo capsules	3 months parallel design	P-F Study; Stroop; Dementia-detecting test	*Treatment > placebo on extragression (increased in placebo group during exam time);* treatment = placebo on other measures
Gesch *et al*. (2002) [150]	*N = *172 (male offenders > 18 years); prison inmates	Vitamin and mineral supplement + 80mg EPA, 44mg DHA, 1.26g ALA or veg oil placebo	20 weeks, parallel design	No. disciplinary actions; GATB; ECQ; SAS; HADQ	*Treatment > placebo on OAS-M & MADRS*
Zanarini *et al*. (2003) [149]	*N* = 30 (18-40 years; *M* = 26); females with moderately severe borderline personality disorder	1g/day ethyl-EPA or mineral oil placebo	8 weeks parallel design	OAS-M; MADRS	*Treatment > placebo no. disciplinary actions *
					Treatment = placebo on psychological measures
Bradbury *et al.* (2004) [148]	Stressed university staff (PSS scores ≥ 17) ISRCTN22569553	1.5g DHA + 360mg EPA + Vit E, olive oil placebo, or no treatment control	6 weeks parallel design	PSS	Treatment = placebo on PSS; *treatment > no treatment control*
Buydens-Branchey *et al.* (2008) [156]	*N *= 24 (mean age 51 years); patients with history of aggression	2.5g EPA + 0.5g DHA/day + Vit E or soybean oil + Vit E placebo	3 months parallel design, most taking medication	Anger score on POMS	*Treatment > placebo on POMS anger scores*
Yehuda *et al.* (1996) [168]	*N =* 100 (50-73 years; 21% females) AD patients	0.5g ALA :LA, 1 :4 ratio (n = 60) or mineral oil placebo	4 weeks parallel design, adjunctive therapy	12-item quality of life questionnaire (caregiver), clinician interview	*Treatment > placebo on 12-item QOL questionnaire. *
					Clinician interviews not reported.
Terano *et al.* (1999) [169]	*N* = 20 (mean age 83) nursing home residents with mild-moderate vascular dementia	4.32g/day DHA or ‘control’	12 months parallel design	MMSE; HDS-R; clinical evaluation	*Treatment > placebo on outcome measures after 3 & 6 months, associated with DHA increases*
Kotani *et al.* (2006) [171]	*N = *21 (mean age 68; 57% male); outpatients with MCI	240mg/day AA+DHA or olive oil placebo	3 months parallel design	RBANS (Japanese version)	*Treatment > placebo on immediate memory & attention. *Treatment = placebo on other 10 sub-scales
Freund-Levi *et al.* (2006) [172]	*N *= 178 (mean age 74); mild-moderate AD patients NCT00211159	1.72g DHA + 600mg EPA/day or placebo	6 months parallel design, adjunctive therapy	MMSE, ADAS-cog; global function on ^b^CDRS	Treatment = placebo on outcome measures. *Treatment > placebo on MMSE in mild MCI group (n = 27)*
Chiu *et al.* (2008) [173]	*N* = 35 (mean age 74; 57% female); AD or MCI	1.08g EPA + .72g DHA or olive oil placebo	6 months	CIBIC-plus; ADAS-cog; MMSE; HDRS	*Treatment > placebo on CIBIC-plus*. Treatment = placebo on other measures. *Treatment > placebo on ADAS-cog in MCI sub-group *
van de Rest *et al.* (2008) [174]	*N* = 302 (mean age 70 years; 55% male) community non-demented dwelling adults NTR97; ISRCTN46249783	1.8g/day EPA+DHA; 400mg/day EPA + DHA; or sunflower oil placebo; tocopherol added	26 weeks parallel design	Word Learning Task; Digit Span; Trail Making; Stroop; Verbal Fluency	Treatment = placebo on outcome measures; *Treatment > placebo on attention for apoE-4 carriers and males*

*Note*: Trials are grouped according to subheadings in main text (e.g., ADHD, depression, etc.) *Positive treatment effects are presented in italic. AD = Alzheimer’s Disease; ADAS-cog = cognitive portion of the Alzheimer’s Disease Assessment Scale; ADHD = attention deficit hyperactivity disorder; AIMS = Abnormal Involuntary Movement Scale; ASQ = Conners’ Abbreviated Symptom Questionnaires; BDI = Beck Depression Inventory; BPRS = Brief Psychiatric Rating Scale; CBC = Child Behavior Checklist; CCT = Children’s Color Trails test; CDI = Childhood Depression Inventory; ^a^CDRS = Childhood Depression Rating Scale; ^b^CDRS = Clinical Dementia Rating Scale; CDSS = Calgary Depression Scale for Schizophrenia; CES-D = Centre for Epidemiologic Studies Depression Scale; CGB = Caregivers Burden Scale; CGI-S = Clinical Global Impression-Severity; CIBIC-plus = Clinician’s Interview-Based Impression of Change Scale; CPRS = Conners’ Parent Rating Scales; CPT = Conners’ Continuous Performance Test; CTRS = Conners’ Teacher Rating Scales; DAD = Disability Assessment for Dementia scale; DASS = Depression & Anxiety Stress Scale; DBD = Disruptive Behavior Disorders rating scale; DHUS: Daily Hassles & Uplifts Scale; DMT: Delayed Memory Task; DSM-IV = Diagnostic and Statistical Manual of Mental Disorders, version 4; DTVP = Development Test of Visual Perception; ECQ = Emotional Control Questionnaire; EPDS = Edinburgh Postnatal Depression Scale; ESRS = Extrapyramidal Symptom Rating Scale; FADS = fatty acid deficiency symptoms; GAFS = Global Assessment of Functioning Scale (revised GAS); GAS = Global Assessment Scale; GATB = General Aptitude Test Battery; GDS = Geriatric Depression Scale; HADQ = Hospital Anxiety & Depression Questionnaire; HDRS = Hamilton Depression Rating Scale; HSCL-D-20 = 20-item Hopkins Symptom Checklist Depression Scale; IDS = Inventory for Depressive Symptomology; IMT = Immediate Memory Task; LUNSERS = Liverpool University Neuroleptic Side-Effects Rating Scale; MABC = Movement Assessment Battery for Children; MADRS = Montgomery-Asberg Depression Rating Scale; MCI = Mild Cognitive Impairment; MMSE = Mini-Mental State Examination; NPI = Neuropsychiatric Inventory; OAS-M = The Overt Aggression Scale, Modified; PANSS = Positive and Negative Syndrome Scale; PGWB = Psychological General Well-Being Schedule; P-F Study: measures aggression, including extra- & intra-aggression; POMS = Profile of Mood States; PSS: Perceived Stress Scale; RBANS = Repeatable Battery for the Assessment of Neuropsychological Status; SANS = Scale for the Assessment of Negative Symptoms; SARS = Simpson-Angus Rating Scale; SAS = Survey Anger Scales; SOFAS = Social and Occupational Functioning Assessment Scale; STAEI = State-Trait Anger Expression Inventory; Stroop = Stroop color-word test; STM = Short-term memory; TEA-ch = Test of Everyday Attention for children; TOVA = Test of Variables of Attention; Vit E = Vitamin E (α-tocopheryl acetate); WISC-III = Wechsler Intelligence Scale for Children, version 3; WORD = Wechsler Objective Reading Dimensions; WJPEB-R = Woodstock-Johnston Psycho-Educational Battery – Revised; YMRS = Young Mania Rating Scale

### 3.1. Attention Deficit Hyperactivity Disorder (ADHD)

Increasing attention has been given to the role of EFA in childhood developmental disorders. Given that brain development continues throughout childhood [42,43], PUFA supplementation may also have a beneficial effect during developmental milestones, particularly in children with developmental disorders that might be related to problems with PUFA metabolism and/or deficiency. 

Since the 1980s, researchers have identified lower levels of n-3 and n-6 PUFA in blood analyses of hyperactive children compared with matched controls [44,45,46,47,48,49,50,51]. Therefore a growing number of clinical trials are investigating effects of PUFA supplementation on ADHD-related symptoms.

Early intervention trials were reported to show little or no improvement in alleviating hyperactive symptoms of ADHD with evening primrose oil, which contains the n-6 PUFA GLA [44,52]. Following these studies and with increasing awareness of the importance of n-3 PUFA, 8 randomised, double-blind, placebo-controlled PUFA supplementation trials have been completed with children displaying ADHD-related symptoms, with varying results. These could be attributed to methodological differences such as variations in dosage, PUFA formulation, period of supplementation, selection criteria and outcome measures.

In the first study, supplementation with 345mg of DHA per day over four months in 6–12 year old children diagnosed with ADHD did not significantly improve objective or subjective measurements of symptoms, even though there was a significant increase in RBC levels of DHA in the DHA group [53]. In this study, stimulant medication ceased 24 hours before laboratory measurements. However, parents completed the subjective scales (Child Behavior Checklist and Conners’ Rating Scales) while the children were still receiving stimulant medication. This may have influenced the results by masking their symptoms and making it difficult to detect any improvements; closer inspection confirms that t-scores on parent rating scales were in the normal range before supplementation. Moreover, children who had experienced ineffective treatment with stimulant medication or had a previous diagnosis of mood, anxiety, thought or bipolar disorder were excluded from the study. It is presumed that the rationale for this was to select a group of children with ‘pure’ ADHD symptoms. This may not be entirely practical due to the high co-morbidity prevalent in ADHD and, as discussed by Richardson [54], the heterogeneity of ADHD and its overlap with other neurodevelopmental disorders needs to be explored within a phospholipid framework.

Another study using both DHA and EPA (supplemented in fish oil-enriched bread, supplying 3600mg DHA and 700mg EPA per week, or 514mg DHA and 100mg EPA per day) [55] also found no significant treatment effects on ADHD symptoms, in a two month placebo-controlled, double-blind trial with 40 children aged 6-12 who were mostly drug-free (34/40). The placebo bread contained olive oil. Paradoxically, the control group had significant improvements in visual short-term memory and errors of commission that were not seen in the treatment group. Blood samples were not taken, so it is not clear whether this sample had low n-3 PUFA levels at baseline. Given that the study was conducted in Japan, known to have high fish consumption, it is likely that baseline levels were relatively high. It is also possible that two months may have been insufficient time for effects to become observable, as it may take longer to elevate the fatty acid composition of neural membranes [56,57]. 

Five other interventions reported improvements in symptoms with a combination of n-6 and n-3 PUFA, including both EPA and DHA. In a pilot study, 41 British children aged 8–12 years with ADHD-related symptoms and specific learning disabilities (dyslexia) were given a PUFA supplement or olive oil placebo over 12 weeks [58]. The PUFA supplement in this study provided a daily dose of 186mg EPA, 480mg DHA, 864mg LA and 42mg AA, as well as α-tocopherol (Vitamin E). These children all had above average scores on parent ratings of ADHD symptoms, and average general ability yet low reading achievement. Baseline measures on learning and behaviour did not differ between the groups, but the PUFA group showed improvements on global ADHD indices and subscale ratings of cognitive problems/inattention, hyperactivity, restlessness/impulsivity, psychosomatic complaints and anxiety/shyness on Conners’ Parent Rating Scale following 12 weeks of supplementation, with medium effect sizes reported.

Similarly, another study investigated effects of PUFA supplementation, providing a daily dose of 80mg EPA, 480mg DHA, 40mg AA, 96g GLA and 24mg α-tocopherol, or olive oil placebo, over four months in American children with ADHD-like symptoms, selected for thirst and skin problems indicative of fatty acid deficiency [59]. Plasma proportions of DHA, EPA and α-tocopherol increased in the intervention group following supplementation and there was a significant drop in n-6 to n-3 ratios, although the olive oil group demonstrated a small increase in ALA. Significant treatment effects were found for only 2 of 16 outcome measures when compared to placebo: parent ratings of conduct and teacher ratings of attention. Oppositional defiant behavior scores significantly improved from clinical to non-clinical levels, and significant relationships were reported between the change in RBC PUFA and the magnitude of improvements in outcome measures. Increased levels of EPA and DHA were associated with decreases in teacher ratings of inattention and increased levels of EPA with parent ratings of reduced disruptive behaviour. Methodological issues in this study included increased n-3 plasma levels in the placebo (olive oil) group and small sample size (*N* = 50 plus 17 dropouts). 

Two larger double-blind, placebo-controlled trials investigated effects of PUFA on ADHD symptoms. The first study [60] included 117 children diagnosed with dyspraxia, or Developmental Coordination Disorder (DCD), and a third of the sample had scores Ź 2 SD above the population mean on the DSM-IV ADHD subscale of Conners’ Teacher Rating Scales (CTRS). The children were functioning on average about a year behind their chronological age in reading and spelling. The active supplement provided a high ratio of EPA:DHA with a daily dose of 552 mg EPA, 168 mg DHA, 60mg DGLA and 9.6 mg α-tocopherol. Although no improvements were detected on the core DCD symptom of poor motor function after three months, highly significant improvements were reported in reading and spelling in the treatment group compared to placebo over three months and significant treatment effects were observed for teacher ratings of oppositional behaviour, cognitive problems/inattention, hyperactivity, anxiety/shyness, and global/DSM-IV ADHD. Following a one-way, uncontrolled crossover to active treatment for a further three months, the placebo group demonstrated similar improvements in mean scores, while the original treatment group’s scores continued to improve. 

The second large intervention included 132 Australian children, all with ADHD symptoms > 90^th^ percentile and therefore in the clinical range for a diagnosis [61]. Children received the same treatment as above (a daily dose of 552 mg EPA, 168 mg DHA, 60mg DGLA and 9.6 mg α-tocopherol) with or without a micronutrient supplement or placebo for 15 weeks, and then all groups crossed over to active treatment for a further 15 weeks. Significant improvements were observed on parent ratings of inattention, hyperactivity and impulsivity in both PUFA groups over 15 weeks, which were then mirrored in the placebo group after switching to active treatment for 15 weeks. Objective improvements were found in children’s ability to switch and control their attention, and on vocabulary scores, and these mediated parent-reported improvements in hyperactivity, impulsivity and inattention [62]. This study also found that the fatty acid deficiency symptoms used as a selection criterion by Stevens *et al. *[59] improved in both the treatment and placebo groups over 15 weeks, and concluded that these symptoms are more likely to be an indicator of n-6 rather than n-3 deficiency [63].

A more recent study was conducted in Sweden with a similar supplement and study design as the previous two large trials, but including 75 children aged 8-18 years with ADHD [64]. They did not detect significant improvements overall; however, when dividing the sample into subgroups, they found that children with the inattentive subtype of ADHD and associated neurodevelopmental disorders, *i.e.* autistic symptoms and learning difficulties, showed similar clinical improvements as the previous large studies.

Therefore, there is evidence that children with ADHD-related symptoms may respond to PUFA supplementation and that those with the attention subtype and/or learning difficulties may be more likely to respond. Interestingly, studies using a combination of n-3 and n-6 fatty acids have been more effective than those with DHA or GLA (DGLA precursor) alone. However, the relative importance of EPA, DHA and GLA remain to be determined due to methodological differences between the studies. Our group has recently completed a 12 month trial with 7–12 year old children with ADHD in Adelaide and Brisbane, Australia, in a 3 x 3 crossover design comparing EPA-rich and DHA-rich oils with an LA placebo. At baseline higher DHA was associated with improved reading (although not quite significant after controlling for confounders) and higher AA with poorer reading, spelling, vocabulary and attention. Interestingly, children with learning difficulties (behind age level on literacy; *n* = 30 with bloods) had lower DHA and total n-3 and higher n-6 PUFA levels, although only the DHA relationship remained significant after controlling for differences in age and parent-reported health [65]. Preliminary results indicate that, although there were no significant between-group differences over 4-months, in children who consented to have blood taken increased erythrocyte DHA was associated with improved parent ratings of oppositional behaviour, anxiety/shyness, divided attention and reading (*n* = 45). In the subgroup with learning difficulties (*n* = 16 with bloods at both time points) increased erythrocyte DHA had even stronger associations with improved parent ratings of oppositional behaviour, hyperactivity, restlessness/impulsivity, divided attention, reading and spelling [66]. These preliminary data indicate that DHA supplementation may be associated with improved ADHD symptoms and that children with learning difficulties may be more likely to benefit, supporting a phospholipid spectrum hypothesis that suggests these symptoms occur on a continuum of overlapping and related developmental difficulties [54]. However, the latter sample size was small due to difficulties with recruitment/eligibility and need to be investigated further in larger samples.

Other randomized controlled trials with ADHD and n-3 PUFA are underway in Israel, the UK and France, as listed in Table 2.

### 3.2. Other Developmental Disorders

PUFA have also been implicated in other, often related developmental disorders such as autistic spectrum disorder [67,68] and dyslexia [69,70,71,72,73,74]. There is some evidence to suggest that autism may involve functional deficiencies or imbalances in n-3 PUFA. Studies on the profiles of PUFA in children with autism that have found defects of PUFA and phospholipids, the major constituent of cell membranes, have had conflicting results. Vancassel *et al.* examined plasma phospholipid fatty acids in a population of children with autism compared to intellectually disabled controls [75]. They reported a 23% reduction of DHA levels in the children with autism compared with controls. Reduced RBC levels of n-3 PUFA and increased levels of saturated fatty acids (SFA) have also been reported in individuals with autism [67,68]. Additionally, there is evidence that n-3 PUFA can assist in inflammatory bowel disorders [76], which are often found in children with autism and may play a role in its etiology [77].

Few studies have investigated effects of n-3 PUFA supplementation in autism. A case report noted improvements in symptoms of a boy with autism following EPA supplementation that was increased from 1g per day to 3g per day over four weeks, with improvements continuing after an eight-month follow-up [78]. An uncontrolled open-label study completed by 18 out of 20 children reported significant within-group improvements after three months of combined n-3, n-6 and n-9 PUFA supplementation (1g/day) [79]. Only one randomized controlled trial (RCT) trialing n-3 PUFA supplementation in autism has been published [80]. This six-week pilot RCT in 13 children with autism reported a trend for improvements in hyperactivity and stereotypy in the group receiving n-3 PUFA. Menhaden fish oil was used with daily doses of 840 mg EPA and 700 mg DHA. 

A three-month crossover RCT was conducted in Durham by Dr Madeleine Portwood, and is yet to be published. The daily doses of PUFA used were 558 mg EPA and 174 mg DHA. Results regarding outcome measures were inconclusive and postulated to be due to failure to reach a ceiling effect of the PUFA over three months. After a one-way crossover, however, improvements were reportedly observed after six months of active n-3 PUFA supplementation. Therefore it was concluded that further studies examining active versus placebo treatment over six months are warranted. As with previous large studies that extended the treatment period in a one-way crossover [60,61], this study indicates the importance of the length of supplementation relative to dosage for improvements to be observed. Considering the complexity of autism, n-3 PUFA may also be best investigated as an adjunctive therapy. Therefore, studies are limited and results are inconclusive. A study is currently underway in the US investigating n-3 PUFA for treatment of hyperactivity in children with autism and another registered study aiming to investigate n-3 PUFA supplementation in children and adolescents with autistic spectrum disorder aimed to complete in 2007 but does not appear to have published results (see Table 2).

### 3.3. Mental Retardation

To date, fatty acid researchers have not focused specifically on the heterogeneous group of disorders that characterize mental retardation. Many people with mental retardation are likely to display co-morbid symptoms including hyperactivity, impulsivity, aggression, anxiety, and DSM-IV diagnosed psychiatric disorders such as bipolar disorder, schizophrenia [81] and ADHD [82]. It has been suggested that the high rate of psychiatric disorders in children and adolescents with mental retardation might be a major problem in their functioning and adaptation [83]. The impact of PUFA for the amelioration of learning, cognition and behavior control problems in developmental disorders like ADHD may have implications for the treatment of these same difficulties in people with mental retardation. This is important, particularly given that there are indications that some forms of mental retardation might be caused by defects in the structure and functioning of neural synapses [84], which are rich in the n-3 PUFA, DHA. For a more in-depth discussion see Sinn and Wilson [85].

### 3.4. Depression and Suicide Ideation

PUFA deficiency is also implicated in depression. Along with cardiovascular disease (CVD), depression seems to be more prevalent in countries where fish consumption is lower, after controlling for demographic variables and potentially confounding factors such as alcohol intake [86,87,88,89,90]. It is well established that depression often co-occurs with CVD, which is associated with elevated cholesterol and lower PUFA levels, and studies have confirmed that depression does not result from CVD as it has been found to exist before symptoms of CVD set in [91]. Indicative of a common underlying biological component, higher serum AA:EPA ratios were reported in depressed post-myocardial infarction patients [92] and lower total n-3 PUFA and DHA levels in depressed patients recovering from acute coronary syndromes [93] than in similar patients without depression. Shared PUFA abnormalities might further account for the depression that co-occurs with stress, post-natal depression, autoimmune diseases, multiple sclerosis, cancer, diabetes, aging and osteoporosis [91]. A number of studies have found lower n-3 PUFA levels or higher AA/EPA ratios in people with depression compared with controls [21,94,95,96,97,98,99], including a postmortem comparison of 15 patients with major depressive disorder (MDD) versus 27 controls – specifically lower DHA levels in the frontal cortex [100]. 

A case study reported improvement in treatment-resistant depression when EPA was added to the conventional treatment, which was also associated with reduced neuronal phospholipid turnover and structural brain changes over the nine months of treatment [101]. Clinical trials have been previously reviewed [41,102], although new studies have since been published. Six trials used high EPA supplements. In a small dose-ranging study, treatment with 1g/day of ethyl-EPA but not 2 or 4g/day was effective in improving depressive symptoms in adults with ongoing depression whilst continuing their standard medication [103]. However, another small study reported positive treatment effects with 2g ethyl-EPA as an adjunctive therapy compared with placebo over four weeks [104]. This group also conducted a study with 6-12 year old children with major depressive disorder [105]. Only five of the twenty children were medicated, and following sixteen weeks of 400mg EPA plus 200mg DHA per day, the treatment group showed significant improvements in symptoms on all outcome measures. 

It should be noted that blood samples were not collected in the latter studies. After eight weeks of supplementation with large doses of n-3 PUFA per day (4.4g EPA, 2.2g DHA) as an adjunctive therapy [106], depressed patients in the n-3 PUFA group had significantly lower depression scores than the control group (*p* < 0.001). Importantly, this team did collect blood samples and reported average baseline blood DHA levels of 2.5% of fatty acids in the placebo group and 2.4% in the treatment group (the latter increased to 5.8% in the DHA group after treatment). According to the Omega-3 Index for cardiovascular health the baseline levels correspond with the risk category for CVD (EPA + DHA: 4% of total membrane fatty acids) [11] and are therefore indicative of suboptimal n-3 PUFA levels. There is some recent evidence that 1 g/day EPA may have similar therapeutic effects to 20 mg/day fluoxetine as a treatment for major depression over eight weeks and that using the two treatments in combination is superior to either of them alone [107]. A larger recent study (*N* = 120) found no improvements in depressive symptoms following 1.5g ethyl-EPA plus 0.5g ethyl-DHA supplementation in middle-aged women with psychological distress over eight weeks [109], although they reported improvements in women who did not meet criteria for major depressive episode at baseline. A more recent study investigated treatment with 2.2g EPA and 1.2g DHA in Menhaden fish oil as a monotherapy for major depressive disorder in pregnant women and found significant improvements in the treatment group on all outcome measures compared with placebo [109]. This study is interesting because n-3 PUFA are preferentially transported to the growing fetus during pregnancy, which can deplete n-3 PUFA levels in pregnant mothers. An uncontrolled study also reported reductions in postpartum depression following n-3 PUFA supplementation over eight weeks [110].

Four studies using DHA or DHA-rich oils did not report improvements following n-3 supplementation. One of these studies supplemented patients with 2g DHA for only six weeks as a monotherapy [111]; one supplied 0.6g EPA + 2.4g DHA as an adjunctive therapy for 12 weeks [112] and the largest study gave a similar dosage as an adjunctive therapy for 16 weeks [113]. It should be noted though that two of these studies collected blood samples and revealed normal baseline n-3 levels; Marangell *et al. *[111] reported that the DHA group and placebo group had 4.15% and 3.78% DHA as a percentage of total fatty acids at baseline, respectively, and Grenyer *et al. *[113] reported baseline DHA as 4.2% of total RBC fatty acids. Although in the latter study reductions in depression scores did not differ between the groups, both treatment and placebo groups had received counseling, which is likely to account for improved mood in the whole sample. Furthermore, these authors later reported that increased DHA levels were significantly associated with improvements in depression scores [114], warranting further research into DHA and depression. Therefore studies using high EPA supplements should also determine to what extent the EPA has been converted to DHA and which of these is associated with improvements. 

A larger study investigated depressive symptoms in a cohort of elderly people with mild-moderate Alzheimer’s disease who were living in their own homes. This study (cognitive results reported further below) found no benefits of treatment on neuropsychiatric symptoms overall, although improved mood was detected in the treatment group compared with placebo in non apoE-4 allele carriers and reduced agitation in apoE-4 carriers [115]. Given the incidence of depression in Alzheimer’s disease, and increased risk of depressed people developing dementia, this merits further investigation. Another recently published study investigated additive effects of supplementation with ethyl ester EPA and DHA on depressive symptoms in conjunction with sertraline in patients with major depression and coronary heart disease, finding no benefits of treatment over placebo [116].

In one of two further, larger population based studies, Rogers *et al.* randomized a community sample of 190 individuals with mild to moderate depression to a combined EPA + DHA supplement (1.5g per day) or olive oil placebo for 12 weeks [117]. They did not report any favourable effects of the supplement on depressive symptoms or a range of outcomes and concluded that this supports a negligible effect of n-3 on depression. A group from the Netherlands also found no effect of n-3 PUFA supplementation on mood in 302 independently living adults [118]. These studies did not use the Hamilton Depression Rating Scales (HDRS), which has been generally used as a primary outcome measure by the other studies, thereby reducing comparability. Although Rogers *et al.* took blood samples, they measured levels in plasma rather than RBC, and it should be noted that other studies reporting improvements in depression scores tended to use inpatients with more severe depression, many of whom were treatment resistant. Finally, some studies used a placebo lead-in phase to eliminate placebo responders [106], and others used patients who had depression scores indicating impaired function despite ongoing treatment with medication [103]. These selection criteria also could have influenced the outcomes: eliminating placebo responders may be pertinent to clinical trials in depression because often depressive symptoms can lift due to the attention associated with taking part in a clinical trial; non-responders to medication may be a subgroup more likely to respond to n-3 PUFA. Interestingly, Carney *et al.* did use a two-week placebo lead in phase but did not include non-responders to medication, and improvements were seen in both treatment and placebo groups, possibly in response to the sertraline administered as an adjunctive therapy [116].

In summary, 13 randomized controlled trials that investigated effects of n-3 PUFA on depressive symptoms were identified, ranging from 4–26 weeks with sample sizes ranging from *N* = 20–302, in wide-ranging populations including psychiatric patients, coronary patients, pregnant women, elderly people with Alzheimer’s disease and non-depressed community cohorts, one with children, some as adjunctive therapy to existing medication, using variable n-3 PUFA dosages, ratios of EPA:DHA and outcome measures. Five studies reported improvements overall, and two in subgroup analyses. Clearly more studies are needed with larger sample sizes and attention to selection criteria and likely responders, baseline n-3 PUFA levels, erythrocyte blood samples, length of supplementation and comparison of EPA *vs.* DHA-rich oils. A large number of randomized controlled trials are currently underway around the world investigating n-3 PUFA in depression, including two US studies that are comparing EPA and DHA – see Table 2 for a summary. 

### 3.5. Bipolar Disorder

In addition to finding lower DHA levels in the frontal cortex of patients with MDD, McNamara *et al.* also identified lower DHA levels in the postmortem frontal cortex of 18 patients with bipolar depression compared with 19 age-matched controls [119]. One of the pioneering intervention trials reported on n-3 fatty acids and psychopathology was conducted by Stoll *et al. *[120] in bipolar disorder patients. The placebo-controlled, double-blind clinical trial produced a significantly greater response to treatment (64%) in the n-3 PUFA group than in those given olive oil placebos (18.8%). In this study, patients received fish oil capsules containing a total of 6.2g EPA and 3.4g DHA daily over four months, with no adverse effects overall. Since then another five studies have been conducted with bipolar patients. An open label study (*N* = 12) provided 1.5-2g ethyl EPA for 24 weeks as an adjunctive therapy [121]. Although there was no control group, at one month 7/10 of these previously treatment-resistant patients showed ≥ 50% reduction in HDRS scores and scores in five completers were dramatically reduced at six months. The other placebo-controlled studies provided ethyl EPA as an adjunctive therapy, supplying 1-2 g for 12 weeks [122] and 6g for 16 weeks [123]. Keck *et al.* reported no improvements in symptoms but Frangou *et al. *[122] reported significant improvements with 1g and 2g/day ethyl EPA over placebo in two out of three outcome measures. None of these studies took blood samples. 

Frangou *et al. *[124] also reported increased brain levels of N-acetyl-aspartate (NAA), a presumed marker of neuronal integrity with 2g ethyl-EPA in 14 female outpatients compared with placebo, although this was not associated with improvements in symptoms. Treatment with n-3 PUFA in an uncontrolled study with a small group of women with bipolar disorder found no significant improvement over four weeks but also reported alterations in a marker of membrane fluidity in the n-3 groups compared with bipolar patients not taking n-3 PUFA and healthy controls [125]. These alterations in markers of brain function should be explored further, and perhaps in the context of multimodal approaches to treatment.

Depression and bipolar disorder are often associated with suicidal ideation. The relationship between PUFA, increased suicide risk and related disorders is considered in a review by Brunner and colleagues [126], who report an association between low cholesterol and higher risk of suicide. A recent study investigated n-3 PUFA supplementation, in addition to standard psychiatric care, on psychometric measures in adults with recurrent non-fatal self harm [127]. Daily supplementation with n-3 PUFA (1.2 g EPA and 0.9 g DHA) for 12 weeks improved psychopathology, specifically scores for depression, suicidality and daily stresses, compared with placebo in 49 participants.

In summary, two controlled studies have improved bipolar symptoms with a large dose combination of EPA and DHA over four months and 1-2g/day ethyl-EPA over 12 weeks, and there is some evidence from another study that this may impact on suicidal ideation. Again, further investigation of supplement formulation and blood analyses of erythrocyte n-3 PUFAs are needed to investigate relative efficacy of EPA *vs.* DHA *vs.* a combination of the two. Randomized controlled trials in bipolar depression are currently underway in Germany and the US (see Table 2).

**Table 2:** Registered (unpublished) double-blind randomized placebo-controlled trials of omega-3 fatty acids in mental illness.*

Registrant	Country	Title of study	Date registered	Registration number
Eric Taylor, Child & Adolescent Institute of Psychiatry	UK	Omega-3 fatty acid supplementation for adolescent boys with ADHD: a double-blind, randomized controlled trial (MAAFA)	09/05/2006	ISRCTN27741572
Natalie Sinn; University of South Australia & Queensland University of Technology	Australia	Randomized controlled trial investigating effects of supplementation with omega-3 fatty acids EPA and DHA versus omega-6 fatty acid LA on ADHD symptoms and learning difficulties in 7-12 year old children	20/06/2007	ACTRN 012607000332426
Madeleine Portwood; Durham County Council	UK	The Middlesbrough study: A randomized, controlled trial of dietary supplements with omega-3/omega-6 fatty acids in mainstream school children	28/09/2007	ISRCTN12286781
Laboratories URGO	France	A randomized, controlled, double blind placebo trial to evaluate the efficacy and the tolerance of an omega-3 fatty acids supplement in ADHD children	09/10/2008	NCT00770627
Hadassah Medical Organisation	Israel	The effect of omega-3 fatty acid supplementation on behavior of children with ADHD	01/04/2009	NCT00874536
Sherie Novotny; New Jersey University of Medicine and Dentistry	USA	Omega-3 fatty acids in the treatment of children with autism spectrum disorders	27/04/2007	NCT00467818
Stephen Bent; University of California	USA	A randomized, double-blind, placebo-controlled 12-week study to investigate the effect of omega-3 fatty acids on hyperactivity in childhood autism	05/11/2008	NCT00786799
Atul Singhal, Institute of Child Health	UK	The influence of n-3 fatty acid supplementation on vascular and cognitive function in healthy young adults; a randomized controlled trial	05/05/2005	ISRCTN19987575
Lev Gertsik; National Center for Complementary and Alternative Medicine	USA	PUFA augmentation in treatment of major depression	14/08/2003	NCT00067301
Maria Makrides; Child Nutrition Research Centre	Australia	A randomized trial of DHA in pregnancy to prevent postnatal depressive symptoms and enhance neurodevelopment in children: The DOMInO Trial	30/09/2005	ACTRN 12605000569606
Anne Marie Rees; University of New South Wales	Australia	A randomized, double-blind, placebo controlled trial of omega-3 polyunsaturated fatty acids as a monotherapy for major depression	11/10/2005	NCT00238758
William Coryell; University of Iowa	USA	Essential fatty acids in management of major depressive Disorder – A pilot study	16/11/2005	NCT00256412
F Pouwer; VU University Medical Centre, EMGO-Institute	Netherlands	Addition of eicosapentaenoic acid to maintenance anti-depressant therapy in diabetes patients with major depressive disorder: a double-blind, placebo-controlled study	21/03/2006	NTR624; ISRCTN30877831
Gordon Parker; University of New South Wales	Australia	A randomized, double-blind, placebo controlled trial of omega-3 polyunsaturated fatty acid as an augmenter of antidepressant medication for major depression	07/02/2006	NCT00289484
Vilma Gabbay; National Centre for Complementary and Alternative Medicine	USA	Omega-3 fatty acids in adolescent depression	07/04/2006	NCT00312897
David Mischoulon; Massachusetts General Hospital	USA	Omega-3 fatty acids for treatment of major depression: Differential effects of EPA and DHA, and associated biochemical and immune parameters	04/08/2006	NCT00361374
Elad Schiff; Bnai Zion Medical Center	Israel	Prevention of depression with omega-3 fatty acids in chronic carriers of hepatitis C treated with interferon alpha	05/12/2006	NCT00408304
Robert McNamara; University of Cincinnati	USA	Evaluation of omega-3 fatty acids as a treatment-adjunct in adolescent patients with major depressive disorder exhibiting partial response to SSRI medication: an open-label neuroimaging trial^a^	03/08/2007	NCT00511810
Danit Shahar; Beersheva Mental Health Center	Israel	Folic acid and omega-3 fatty acid supplementation in depressed older adults - factorial assignment	28/05/2007	NCT00480207
Mark Rapaport; National Institute of Mental Health (NIMH)	USA	Omega-3 fatty acids for treatment of major depression: Differential effects of EPA and DHA, and associated biochemical and immune parameters	14/08/2007	NCT00517036
Matthew Muldoon; University of Pittsburgh	USA	Evaluating the effects of omega-3 fatty acids on heart disease and behavior; Biobehavioral studies of cardiovascular disease	18/04/2008	NCT00663871
Sayed Naqvi; Cedars-Sinai Medical Centre	USA	Omega-3 fatty acids for treatment of depression in adolescents	08/04/2008	NCT00658476
Alexandra Parker; Orygen Youth Health - Research Centre	Australia	The acceptability and effectiveness of a combination of problem solving therapy, behavioral exercise intervention and omega-3 supplements compared to a combination of supportive counseling, exercise psychoeducation, and placebo omega-3 in reducing depression and anxiety symptoms in help-seeking young people aged 12-25 years: A factorial randomized controlled trial	30/10/2008	ACTRN 12608000550303
Geoffrey Schrader, Queen Elizabeth Hospital; Peter Howe, University of South Australia	Australia	Omega-3 fatty acid supplementation for symptoms of depression in patients with cardiovascular disease	01/12/2008	ACTRN 12608000598381
National Science Council	Taiwan	The effect of fish oil in major depressive disorder: a double-blind placebo-controlled monotherapy trial to demonstrate the therapeutic and preventive effects of [sic]depression	31/12/2008	NCT00816322
Vlima Gabbay; NYU School of Medicine	USA	The role of omega-3 fatty acids in adolescent depression	19/08/2009	NCT00962598
Krista Lanctôt; Sunnybrook Health Sciences Centre	Canada	Treating depression in coronary artery disease with omega-3 fatty acids (CAROTID)	10/09/2009	NCT00981383
National Institute of Mental Health (NIMH)	USA	Omega-3 fatty acids in the treatment of major depression and bipolar disorder: A double-blind, placebo-controlled trial	03/11/1999 (estimated completion 2002)	NCT00001146
Andrew Stoll; National Center for Complementary and Alternative Medicine	USA	Omega-3 fatty acids in bipolar disorder prophylaxis	02/02/2001 (estimated completion 2004)	NCT00010868
Barbara Gracious; University of Rochester	USA	A comparison of omega-3 fatty acids *vs.* placebo in children and adolescents with bipolar disorder	09/11/2005	NCT00252486
Janet Wozniak; Massachusetts General Hospital	USA	A randomized placebo controlled clinical trial of omega-3 fatty acid adjunctive to open-label aripiprazole for the treatment of bipolar disorder in children and adolescents ages 6-17 with bipolar spectrum disorder	28/12/2007	NCT00592683
Sencan Unal; Mayo Clinic	USA	Neurometabolic effects of the essential polyunsaturated fatty acids in early-onset bipolar disorder: A magnetic resonance spectroscopy study	21/12/2007	NCT00586222
Emanuel Severus; University of Munich	Germany	Omega-3 fatty acids in bipolar patients with a low omega-3 index and reduced heart rate variability: The BIPO-3 Trial	28/04/2009	NCT00891826
Beth Murphy; Mclean Hospital	USA	A combination of cytidine and omega-3 fatty acids in bipolar disorder: Are there additive or synergistic mood stabilizing effects?	27/02/2009	NCT00854737
Melissa DelBello, Robert McNamara; University of Cincinnati	USA	Neurochemical effects of omega-3 fatty acids in adolescents at risk for mania	08/06/2009	NCT00917501
Jeffrey Yao; University of Pittsburgh	USA	Coronary artery disease risk in schizophrenia: Effect of omega-3 fatty acid supplementation	09/09/2005	NCT00167310
Havard Bentsen; University Hospital, Aker	Norway	A multicentre, placebo-controlled trial of eicosapentaenoic acid (EPA) and antioxidant supplementation in the treatment of schizophrenia and related disorders	05/01/2007	NCT00419146
Neil Richtand; University of Cincinnati	USA	Randomized, double-blind, placebo-controlled pilot trial of essential fatty acid deficiency replacement in early schizophrenia	28/12/2007	NCT00585390
Paul Amminger, Patrick McGorry; Orygen Research Centre	Australia, Austria, China, Denmark, Germany, Switzerland, UK	The NEURAPRO-E (North America, EURope, Australia PROdrome) Study: A multicenter randomized controlled trial of omega-3 fatty acids and cognitive-behavioral case management for symptomatic patients at ultra-high risk for early progression to schizophrenia and other psychotic disorders to assess the 6-month transition rate to first episode psychosis	23/09/2008	ACTRN 12608000475347
Laure Buydens-Branchey; National Institute on Drug Abuse (NIDA)	USA	Effects of fatty acid supplementation on substance dependent individuals	06/04/2006	NCT00312455
Miquel Casas; Hospital Universitari Vall d’Hebron Research Institute	Spain	Efficacy of omega-3 fatty acids on borderline personality disorder: A randomized, double-blind clinical trial	16/02/2007	NCT00437099
John Stein; University of Oxford	UK	Nutrition as a modifiable causal factor in anti-social behaviors: A randomized, placebo controlled, double blind trial (PINUP - PrIson NUtrition Project)	06/01/2009	ISRCTN41104834
Joanne Bradbury; Blackmores Ltd, Southern Cross University	Australia	A pilot randomized controlled double blind intervention study of the effects of docosahexaenoic acid (DHA)-rich fish oil compared with olive oil in psychological stress	20/02/2009	ACTRN 12609000124235
Alan Dangour, Medical Research Council (UK) [179]	UK	The OPAL Study: Older People And n-3 Long-chain polyunsaturated fatty acids (target sample: 800 people)	14/07/2004	ISRCTN72331636
Janet Carter; Department of Health; North East London Mental Health Trust	UK	A randomized placebo-controlled trial of polyunsaturated omega-3 fatty acid in the treatment of dementia; a pilot study	30/09/2005	ISRCTN27372325
Joseph Quinn; National Institute on Aging	USA	A randomized double-blind placebo-controlled trial of the effects of docosahexaenoic acid (DHA) in slowing the progression of Azheimer’s Disease	22/02/2007	NCT00440050
Vanessa Danthiir; Commonwealth Scientific Industrial Research Organisation (CSIRO)	Australia	An 18 month study investigating the effects of long chain omega-3 polyunsaturated fatty acids supplementation on cognition and wellbeing in older people (EPOCH)	24/05/2007	ACTRN 12607000278437
Bruno Vellas; Toulouse University Hospital [180]	France	Assessment of the efficacy of omega-3 fatty acids supplementation, multi-domain intervention or their combination on the change of cognitive functions in frail elderly subjects	02/05/2008	NCT00672685
Natalie Sinn; University of South Australia	Australia	Effects of omega-3 fatty acids high in eicosapentaenoic acid (EPA) or docosahexaenoic acid (DHA) versus placebo on cognition and mood in older adults with mild cognitive impairment	03/04/2009	ACTRN 12609000167268
Katia Tanaka; Universidade Federal de Sao Paulo	Brazil	A randomized controlled trial evaluating the effects of the association of ginkgo biloba, omega-3 and physical exercise in memory and executive functions of older people with Parkinson’s disease	22/07/2009	ACTRN 12609000609257
Christine Marx, Durham VA Medical Centre	USA	Omega-3 fatty acids and post traumatic stress disorder	20/03/2008	NCT00644423
Yutaka Mastuoka; Japan Science and Technology Agency	Japan	Phase 2 study of Tachikawa Project for Prevention of Post-traumatic stress disorder with polyunsaturated fatty acid: TPOP-01 Study	23/04/2008	NCT00671489
Yutaka Mastuoka; Japan Science and Technology Agency	Japan	Tachikawa Project for Prevention of Post-traumatic stress disorder with polyunsaturated fatty acid: TPOP-02 Study	01/05/2008	NCT00671099

Trials are grouped according to subheadings in main text (e.g., ADHD, depression, etc.), apart from three new studies in post-traumatic stress disorder. All trial identification numbers were searched in PubMed to check if they had been published yet. *Located on the World Health Organization’s International Clinical Trials Registry Platform Search Portal (http://apps.who.int/trialsearch/)-search terms ‘omega-3’ or ‘n-3 fatty acids’. ^a^This trial was open label and uncontrolled but was included in the table because of physiological assessment of neurological changes in response to omega-3 supplementation

### 3.6. Schizophrenia

There are a number of indications that phospholipid abnormalities may explain biological underpinnings of schizophrenia [26,128,129,130,131]. Low n-3 fatty acid levels in diets or RBC have consistently been found in people with schizophrenic symptoms compared to controls [132,133,134,135,136]. Clinical trials with schizophrenia have reported mostly positive results. In an apparently open-label trial with 20 schizophrenic patients, significant improvements in symptoms were reported after daily supplementation with 10g fish oil over six weeks, and improvements were reported to correlate with increased n-3 PUFA blood levels [137]. Four double-blind controlled trials with EPA supplementation have produced some significant, positive outcomes [138,139,140] although in Peet *et al.* 2002 [140] only with 2g (*vs.* 1 and 4g) ethyl-EPA per day in the subgroup of patients taking clozapine and improvements were also associated with increased levels of AA. 

Another trial found no differences between schizophrenic patients given ethyl EPA and those given placebo over 16 weeks [141]. Horrobin [142] proposed a number of reasons why an effect might not have been obtained, including the possibility that ethyl EPA may not have therapeutic benefits. Hibbeln *et al. *[143] investigated possible confounding variables using the same data set, and found that PUFA status in schizophrenic patients was predicted by smoking status, gender, and dietary intake of n-3 fatty acids. They concluded that phospholipid abnormalities may be an artefact of these variables. However, Emsley *et al. *[138] found significant differences between active and placebo groups on the Positive and Negative Syndrome Scale after controlling for age, gender, length of illness and dietary n-3 intake; further analyses revealed that these improvements were accounted for by improved dyskinesia.

In another recent study [132], schizophrenic patients were matched with healthy controls on a number of factors known to influence PUFA status including age, gender, dietary patterns, smoking and substance abuse. Schizophrenic patients still had significantly lower levels of EPA and DHA, and higher levels of AA than controls. They then gave patients in their study a combination of EPA, DHA and antioxidants (Vitamins E and C) over four months; there was no placebo group. The researchers reported significant within-group improvements on various measures of psychopathology between pre- and post-treatment, which continued for four months after the treatment was discontinued. These results need to be interpreted with caution given equivalent placebo to treatment effects found in other trials of both depression and schizophrenia. Two more recently published studies focused on first-episode psychosis. The first study was with young adults who were provided 2g/day of ethyl-EPA or placebo along with psychotic medication (the majority had been previously unmedicated) [144]. Initial analyses found no treatment effects over 12 weeks; however did find improvements in patients with non-affective psychosis and in those who had had longer duration of untreated psychosis. Treatment effects were significant at 4-6 weeks then matched the placebo group at 12 weeks, indicating that the ethyl-EPA may have improved response to psychotic medication and/or the effects of the medication matched the ethyl-EPA by 12 weeks. The second study aimed to investigate whether 1.3g/day n-3 PUFA supplementation over 12 weeks could reduce the transition of sub-threshold psychosis to first-episode psychotic disorder in adolescents and young adults over 12 months [145]. There was a significantly reduced transition to psychosis in the treatment group (2/41) compared with the placebo group (11/40). A couple of further trials in schizophrenia, one a multicentre intervention involving the latter research group, are currently underway (see Table 2).

### 3.7. Stress, Aggression, Hostility, Impulsivity, Criminal and Antisocial Behavior

A number of studies have indicated a biological basis for aggression and criminal-related behaviour. Low concentrations of cerebrospinal fluid 5-hydroxyindoleacetic acid (CSF 5-HIAA), a marker of cerebral serotonin turnover, have consistently been associated with impulsive, violent, suicidal, hostile and aggressive behaviors, as well as early onset of alcohol dependence and personality disorders [31,146]. Low PUFA status has also been associated with deficient serotonergic neurotransmission [28,29] and it is possible that low CSF 5-HIAA reflects this deficiency. 

A small number of clinical trials have investigated the impact of PUFA supplementation on aggressive behaviour. A double-blind, placebo-controlled study measured effects of daily supplementation with oil containing 49.3% (1.5g) DHA, 6.7% EPA, 7.3% oleic acid, 3.3% AA and other oils for three months on cognitive outcomes and aggression in a group of 41 healthy Japanese students from two universities [147]. The placebo capsules contained small amounts of LA and ALA. No differences between groups were observed on cognitive tests of executive function, but a significant difference was reported in aggression towards others (‘extraggression’) at the end of the study, during a highly stressful final exam period. Extraggression scores significantly increased in the control groups and decreased (though not significantly) in the DHA supplemented groups. Serum blood analyses confirmed a significant increase in EPA and DHA levels in the treatment groups. Therefore three months’ supplementation with n-3 PUFA seemed to assist students in controlling aggression towards others on a psychological test during a period of high stress. Drawing from research on stress-induced hostility and aggression, an Australian group investigated a possible adaptive role of DHA in perceived stress levels of university staff, finding no differences between treatment and placebo but positive effects in the treatment compared with a control group [148].

Another double-blind study tested effects of supplementation with 1g ethyl-EPA daily or placebo over eight weeks on moderate borderline personality disorder, characterized by emotional reactivity and impulsive aggression, in a group of women [149]. The treatment group had a significantly greater decrease in depression and aggression scores at the end of the study. 

A landmark randomized, placebo-controlled trial was undertaken with 231 young adult prisoners to investigate effects of supplementation with micronutrients and essential fatty acids over four-five months on antisocial behavior [150]. The participants were given multi-vitamin/mineral capsules and fatty acid supplement providing n-3 and n-6 fatty acids (1260mg LA, 160mg GLA, 80mg EPA and 44mg DHA daily) or placebo. During the trial, the treatment group received significantly fewer disciplinary actions, and the greatest effect was noted for violent behavior. These studies add an important dimension to research with PUFA in ADHD, which is commonly associated with antisocial behavior symptomatic of conduct disorder and oppositional defiant disorder in childhood [151,152,153] and criminality and antisocial behavior in late adolescence and adulthood [154,155]. This work is currently being continued on a large scale with prisons in the UK, investigating effects of micronutrients and PUFA on anti-social behaviour in a randomized controlled trial over 4 months (‘PINUP’; see Table 2).

Recently, a small randomized controlled trial in 24 men with substance abuse found that supplementation with 3g/day of n-3 PUFAs for three months improved self-reported scores of anger and anxiety compared to a soybean oil placebo [156]. The active group received five capsules per day each containing 450 mg of EPA, 100 mg of DHA and 50 mg of n-3 PUFAs ALA and DPA. Although five participants in both the placebo and active groups were on antidepressant medication during the study, their doses remained stable. Interestingly, the anger ratings remained lower in the active group for three months after supplementation ceased. Each participant’s n-3 PUFA intake was calculated from a diet questionnaire covering the month preceding the study and it was reported that the participants had a daily intake of 148mg LC n-3 PUFAs, only 30% of the ISSFAL recommended intake of 500 mg/day. Furthermore, those participants who reported a history of violent behaviour had a significantly lower LC n-3 PUFA intake compared to those who had no history of this behaviour. After the 3 month treatment with LC n-3 PUFAs, a low anxiety score was associated with an increase in EPA but not DHA, whereas a low anger score was associated more strongly with DHA. This indicates possible variation between the two LC n-3 PUFAs in their effect on psychiatric conditions and warrants comparison of them in further research. 

A randomized controlled trial is currently investigating the role of n-3 PUFA in alleviating psychological stress in university students undertaking exams, and another study in Spain is assessing n-3 PUFA supplementation for symptoms of borderline personality disorder (Table 2).

### 3.8. Dementia and Alzheimer’s Disease

Free radical activity and phospholipid membrane degeneration have been associated with neurodegenerative disorders. With aging, neural membrane fluidity is generally compromised due to an increase in cholesterol, reduced activity level of desaturase enzymes, impaired phospholipid metabolism and increased oxidative stress [20]. Such derangements may be associated with dementia and Alzheimer’s Disease (AD), e.g., autopsied brains of patients who suffered AD showed significantly higher saturated fat and lower PUFA content, particularly DHA, in the hippocampus and frontal lobes compared to aged controls [157], which is consistent with reports of decreased hippocampus size and function in AD patients [20]. 

Prospective cohort/population studies have indicated that higher fish consumption is associated with reduced risk of dementia/AD [158,159,160,161,162,163,164,165]. A single case report of an elderly dementia patient noted clinical improvement over several months of increased fish consumption, which led the author to hypothesise that n-3 PUFA contributed to his improved functioning, although it could have been attributed to numerous other factors associated with admittance to a nursing home [166]. Investigation of n-3 PUFA status in AD patients did find significantly reduced EPA and DHA levels compared to controls, particularly DHA levels which were consistently less than half those in the control group [167]. 

Relatively few controlled studies have investigated effects of n-3 PUFA supplementation on dementia to date. In a short four-week double blind trial with 100 AD patients, a number of improvements in symptoms were reported in 49 of the 60 patients who received an n-3/n-6 supplement comprising 0.5g of ALA and LA in a 1:4 ratio [168]. 

Supplementation with 4.32g DHA daily for one year improved dementia scores of 10 elderly volunteers with vascular dementia compared with 10 controls who did not receive capsules [169]. Another pilot study in 19 participants with mild to moderate AD treated with 1g/day ethyl-EPA for 12 weeks showed no improvement in cognitive functioning, possibly due to the short period of supplementation and/or the nature of the supplement formulation [170]. Supplementation with 240mg/day of DHA and AA improved immediate memory and attention in 12 adults with mild cognitive impairment (MCI) but not eight adults with AD compared to an olive oil MCI placebo group [171]. In the largest study to date with 174 volunteers with diagnosed AD, only those with very mild AD (*n* = 27) showed reductions in cognitive decline after adjunctive supplementation with 1700 mg/day DHA and 600 mg/day EPA for six months and the placebo group improved after switching to n-3 PUFA for a further 6 months [172]. Similarly, a study recently published online also reported improvements relative to placebo in MCI but not AD patients following supplementation with 1.8g/day n-3 PUFA (1080mg EPA, 720mg DHA) over 24 weeks [173]. One population based study investigated the effect of DHA and EPA on cognitive performance of 302 elderly people > 65 years without dementia and detected no treatment effects over 26 weeks, although it did report improvements in attention in apoE-4 carriers and males [174]. The prior studies indicate that early stages of cognitive decline may be optimal for intervention with n-3 PUFA. MCI is considered a risk factor for AD, with an estimated 16-41% of people with MCI converting to a diagnosis of AD each year and up to 64% after two years. It has been suggested therefore that MCI is possibly the earliest stage of detectable AD [175]. Furthermore, major and minor depression often co-occur in MCI [176] and increase the risk of progressing to AD [177]. 

Further investigation of the role of PUFA in dementia is needed, focusing on larger sample sizes, the nature of the supplement (e.g., EPA versus DHA), dosage, length of supplementation, baseline RBC n-3 PUFA proportions, response in ApoE ε4 allele carriers, longer-term outcomes and therapeutic implications. Finally, cardiovascular risk factors for AD have led to recent suggestions that lifestyle factors such as inadequate physical activity and/or n-3 PUFA intake may contribute to reduced cerebral blood flow and blood-brain barrier integrity [37], supporting the notion that one of the primary functions of n-3 PUFA on mental health may be improved circulatory function [36]. This should also be explored in future research.

We are currently collecting data for a six-month study comparing EPA with DHA-rich supplements versus an LA placebo on mood and cognition in elderly people with MCI. Other controlled trials are underway to assess n-3 PUFA supplementation and cognition in healthy older adults and those with dementia (see Table 2 for details).

## 4. Conclusions

There is growing evidence that suboptimal intakes of n-3 PUFA may be associated with psychopathology over the lifespan and include highly prevalent disorders that present a growing public health concern. Most clinical trials have been conducted with major depressive illness in adulthood and in childhood disorders; there are growing numbers of interventions with cognitive decline in older adulthood. Although the causes of these mental health problems are complex and multifactorial, even from a nutritional perspective alone [e.g., 40, 178], dietary and lifestyle factors including n-3 PUFA present modifiable risk factors that can be accessed relatively easily by individuals. 

However, findings of clinical trials have been inconsistent and in many cases inconclusive; methodological differences between studies need to be critically evaluated before drawing conclusions about the efficacy or otherwise of n-3 PUFA in alleviating symptoms. These include sample size, selection criteria, the dosage of the supplement, the nature of the fatty acids (i.e. EPA- or DHA-rich or combined oil; inclusion of n-6 PUFA), and length of supplementation. Given the widespread use of ethyl-esthers, short- and long-term investigation of bioavailability compared with naturally occurring fatty acids in fish oil is warranted. The role of n-6 PUFA also needs to be clarified as some studies found improved and some worse outcomes associated with AA, and successful studies with children included evening primrose oil in the treatment. Some studies in depression have included inpatients with clinical depression despite ongoing medication. Non-responders to other treatments are a possible future area of attention for n-3 PUFA and/or micronutrient supplementation because suboptimal nutrient levels may explain this lack of response to other treatments. Some depression studies have conducted a placebo-lead in phase to eliminate placebo responders and this may also assist in clarifying true treatment effects. Importantly, studies need to identify baseline n-3 PUFA levels in erythrocyte membranes because this may be a major contributor to different findings between studies. This will also assist researchers and clinicians in identifying likely responders. 

Outcome measures also need consideration; as can be seen in Table 1, a great variety of psychological/cognitive outcome measures have been used across these studies and their sensitivity to detecting effects of n-3 PUFA supplementation is varied. Of particular interest are two large studies in the UK [60,150]. In most studies with children, parent and teachers have reported improvements; however cognitive assessments detected varied (mostly no) improvements. In the Durham trial [60], reading and spelling were included as outcome measures with highly significant, and meaningful, improvements in these literacy indicators. In the prison-based trial with criminal offenders [150], a range of psychological assessments were used that did not detect any response to micronutrient/n-3 PUFA supplementation; however prison disciplinary actions were recorded in the treatment and placebo groups and detected significantly less reprimands in the treatment group, particularly in violent behaviour. Therefore selection of outcome measures is an area in serious need of attention.

Finally, only one study appears to have conducted a follow-up; four months after treatment ceased, anger levels remained low [156]; whether this was due to retention of n-3 PUFA in cell membranes or a result of newly learned behavioral changes remains to be determined and also needs to be investigated by future researchers. In conclusion, as well as identifying likely responders, future research investigating effects of n-3 PUFA supplementation on mental health and behavior needs to define meaningful and long term outcomes and optimal supplementation strategies including combinations with other lifestyle risk factors and nutrients that are consumed suboptimally. 

## Notes added in proof

After writing this review another study was identified by a group in Israel (Raz *et al.*, 2009). They published a randomised controlled trial with 7-13 year old unmedicated children with ADHD that reported no treatment effects on parent and teacher questionnaires or a computerised continuous performance task. However the PUFA supplement contained relatively small amounts of LA (480mg) and ALA (120mg), the latter of which is likely to have had minimal conversion to EPA and DHA, and they were only supplemented for 7 weeks, whereas at least 8 and preferably a minimum of 12 weeks tend to be required to show any improvements overall, and with larger doses of long-chain n-3 PUFAs. Interestingly they did report some improvements in both groups which could be a placebo effect although the placebo contained vitamin C and was therefore not a non-active compound.

Raz, R.; Carasso, R. L.; Yehuda, S. The Influence of Short-Chain Essential Fatty Acids on Children with Attention-Deficit/Hyperactivity Disorder: A Double-Blind Placebo-Controlled Study. *J Child Adolesc Psychopharmaco,* **2009**, *19*, 167-177.
